# ^68^Ga-DOTA-D-Alanine-BoroPro Radiotracer for Imaging of the Fibroblast Activation Protein in Malignant and Non-Malignant Diseases

**DOI:** 10.3390/pharmaceutics16040532

**Published:** 2024-04-12

**Authors:** Diana Trujillo-Benítez, Myrna Luna-Gutiérrez, José G. Aguirre-De Paz, Pedro Cruz-Nova, Gerardo Bravo-Villegas, Joel E. Vargas-Ahumada, Paola Vallejo-Armenta, Enrique Morales-Avila, Nallely Jiménez-Mancilla, Rigoberto Oros-Pantoja, Clara Santos-Cuevas, Erika Azorín-Vega, Blanca Ocampo-García, Guillermina Ferro-Flores

**Affiliations:** 1 Department of Radioactive Materials, Instituto Nacional de Investigaciones Nucleares, Ocoyoacac 52750, Mexico; sarahi.t.servicios@inin.gob.mx (D.T.-B.); clara.cuevas@inin.gob.mx (C.S.-C.);; 2 Faculty of Chemistry, Universidad Autónoma del Estado de México, Toluca 50180, Mexico; 3 Nuclear Medicine Department, Instituto Nacional de Cardiología, Mexico City 14000, Mexico; 4 Nuclear Medicine Department, Instituto Nacional de Cancerología, Mexico City 14000, Mexico; 5 Cátedras, CONACyT, Instituto Nacional de Investigaciones Nucleares, Ocoyoacac 52750, Mexico; nallely.jimenez@inin.gob.mx; 6 Faculty of Medicine, Universidad Autónoma del Estado de México, Toluca 50180, Mexico

**Keywords:** fibroblast activation protein, FAP inhibitor, FAP imaging, gallium-68

## Abstract

Recently, we reported a new fibroblast activation protein (FAP) inhibitor radiopharmaceutical based on the ^99m^Tc-((R)-1-((6-hydrazinylnicotinoyl)-D-alanyl) pyrrolidin-2-yl) boronic acid (^99m^Tc-HYNIC-D-Alanine-BoroPro)(^99m^Tc-HYNIC-iFAP) structure for tumor microenvironment SPECT imaging. This research aimed to synthesize ^68^Ga-[2,2′,2″,2‴-(2-(4-(2-(5-(((S)-1-((S)-2-boronopyrrolidin-1-yl)-1-oxopropan-2-yl)carbamoyl)pyridin-2-yl)hydrazine-1-carbothioamido)benzyl)-1,4,7,10-tetraazacyclododecane-1,4,7,10-tetrayl)tetraacetic acid] (^68^Ga-DOTA-D-Alanine-BoroPro)(^68^Ga-iFAP) as a novel radiotracer for PET imaging and evaluate its usefulness for FAP expression in malignant and non-malignant tissues. The coupling of p-SCN-benzene DOTA with HYNIC-iFAP was used for the chemical synthesis and further labeling with ^68^Ga. Radiochemical purity was verified by radio-HPLC. The specificity of ^68^Ga-iFAP was evaluated in HCT116 cells, in which FAP expression was verified by immunofluorescence and Western blot. Biodistribution and biokinetic studies were performed in murine models. ^68^Ga-iFAP uptake at the myocardial level was assessed in mice with induced infarction. First-in-human images of ^68^Ga-iFAP in healthy subjects and patients with myocardial infarction, glioblastoma, prostate cancer, and breast cancer were also obtained. DOTA-D-Alanine BoroPro was prepared with a chemical purity of 98% and was characterized by UPLC mass spectroscopy, FT-IR, and UV-vis. The ^68^Ga-iFAP was obtained with a radiochemical purity of >95%. In vitro and in vivo studies demonstrated ^68^Ga-iFAP-specific recognition for FAP, rapid renal elimination, and adequate visualization of the glioblastoma, breast tumor, prostate cancer, and myocardial infarction sites. The results of this research justify further dosimetry and clinical trials to establish the specificity and sensitivity of ^68^Ga-iFAP PET for FAP expression imaging.

## 1. Introduction

The FAP protein, a serine protease, exhibits overexpression in reactive stromal fibroblasts in more than 90% of epithelial cancers, correlating with a dismal prognosis in colon, pancreatic, ovarian, and hepatocellular carcinomas [1]. In addition, it is present during wound healing and at sites of inflammation such as arthritis and atherosclerotic plaques. However, it is not expressed in healthy tissues [2]. These circumstances have led to the development of specific molecules designed to inhibit FAP (FAPIs) [3]. It has been observed that the inhibition of FAP is not sufficient to achieve a significant change in reducing disease progression [4]. Therefore, various FAPIs are used, primarily with a diagnostic focus and, in some cases, therapeutically when FAPI acts as a carrier for a therapeutic radionuclide or a molecule with pharmacological activity, thereby enhancing treatment efficacy [5].

In the case of cancer, several radionuclide-linked FAP inhibitors have been developed in nuclear medicine. These have been used for both diagnostic purposes, using SPECT (^99m^Tc) [5,6] and PET(^68^Ga, ^18^F) [3,4,5,6,7], and therapy, using ^177^Lu and ^225^Ac [5]. PET and SPECT imaging modalities provide structural and functional insights. However, PET has advantages over SPECT in spatial resolution and sensitivity, with easier metabolic activity quantification and molecular kinetics. However, SPECT is more accessible and less expensive, which is advantageous in certain clinical practices. The choice between PET and SPECT depends on resource availability and clinical needs. While PET/CT and SPECT/CT are typically compared independently, they can be combined to provide complementary data that increases diagnostic accuracy by merging images [8]. FAPI-based imaging is expected to replace other radiopharmaceuticals commonly used in clinical settings. This perspective is due to the potential use of FAPIs as radiotracers in oncologic and non-oncologic diseases, particularly in cardiology, as well as the phenotypic information they can provide for treatment decision making and disease follow-up [9].

The use of FAPI-based radiopharmaceuticals to determine the risk of excessive fibrosis after myocardial infarction has recently been proposed because reactive fibroblasts modulate cardiac fibrosis and, consequently, post-infarction tissue remodeling [10].

Recently, we reported a new fibroblast activation (FAP) inhibitor radiopharmaceutical based on the ^99m^Tc-((R)-1-((6-hydrazinylnicotinoyl)-D-alanyl) pyrrolidin-2-yl) boronic acid (^99m^Tc HYNIC-D-Alanine-BoroPro)(^99m^Tc-HYNIC-iFAP) structure for tumor microenvironment SPECT imaging [7]. In an approach to develop an iFAP ligand for PET imaging, this research aimed to synthesize ^68^Ga-[2,2′,2″,2‴-(2-(4-(2-(5-(((S)-1-((S)-2-boronopyrrolidin-1-yl)-1-oxopropan-2-yl)carbamoyl)pyridin-2-yl)hydrazine-1-carbothioamido)benzyl)-1,4,7,10-tetraazacyclododecane-1,4,7,10-tetrayl)tetraacetic acid] (^68^Ga-DOTA-Benzene-SCN-HYNIC-D-Alanine-BoroPro) (^68^Ga-DOTA-D-Alanine-BoroPro) (^68^Ga-DOTA-iFAP) to evaluate its usefulness for evaluating FAP expression in malignant and non-malignant tissues.

## 2. Materials and Methods

### 2.1. Theoretical Methodology

DOTA-iFAP and FAPI-04 [10] molecular structures were generated in .cdx format using ChemDraw (PerkinElmer, Waltham, MA, USA). Chem3D software (PerkinElmer, Waltham, MA, USA) exported the 3D molecules in .pdb format. Molecular geometries were optimized with a universal force field (UFF) using AVOGADRO 1.2.0 free software (Avogadro: an open-source molecular builder and visualization tool. Version 1.2.0). Then, a second geometry optimization was performed using the free semiempirical MOPAC2016 quantum chemistry software (Stewart Computational Chemistry, free-software licence. Version 2016) with a PM7 theoretical level. The .pdb format was used to export the resulting spatial configuration. 

The Protein Data Bank (PDB) was used to download the three-dimensional structure of the FAP alpha subunit with the code 1Z68. The BIOVIA Discovery Studio 2021 software (Dassault Systèmes, Vélizy-Villacoublay, IdF, France) made adjustments and modifications to the molecule to use this structure in molecular docking calculations. Once these modifications are complete, the resulting structure is in .pdb format. It was then used as a receiver for further analysis. 

AutoDock Tools software (version 1.5.6; The Scripps Research Institute, La Jolla, CA, USA) was employed to generate the receptor configuration as a large molecule and for the ligands FAPI-04 and DOTA-iFAP by generating files with the .pdbqt extension. A search region was defined in the S1 region where amino acid Ser-624 of FAP is located using the XYZ coordinates 18,948, 10,676, 28,989 as the center point and a search box with a size of 90 units on the three axes. The file “AD4_parameters.dat” had to be modified to include the parameters specific to the boron atom, which were obtained from http://mgldev.scripps.edu/pipermail/autodock/2009 March/005439.html (accessed on 17 July 2023). 

The AutoDock software (version 4.2.6; The Scripps Research Institute, La Jolla, CA, USA) evaluated the molecular binding between a protein and ligands. Previously, AutoGrid 4.2.6 was used to generate .map grids. The best affinity complex was then exported in .pdb format, and the log file of molecular binding results was visualized in AutoDock Tools. BIOVA Discovery Studio Visualizer 2021 (Dassault Systemes, Velizy-Villacoublay, IdF, France) was used to inspect and analyze the distances and interactions. Inhibition constants were calculated as previously reported [7].

### 2.2. DOTA-iFAP Synthesis

The iFAP synthesis was carried out as previously reported [7]. For DOTA-iFAP synthesis, 3 mg (4.4 μmol) of DOTA-benzene-p-SCN (Macrocyclics, Dallas, TX, USA) in 0.2 M NaHCO_3_ at pH 9.5 (1 mL) and 2 mg (6.2 μmol) of iFAP peptide were incubated at 95 °C for 2 h. The resulting DOTA-iFAP conjugate was purified by HPLC using a preparative column (C18 column, particle size of 5 μm, length of 2.5 cm; PerkinElmer, Waltham, MA, USA) in online connection with a UV-vis spectrophotometer (Waters Corp., Milford, MA, USA). Linear gradients of 0.1% TFA in water (A) and 0.1% TFA in acetonitrile (B) were applied at 4 mL/min from 100% to 10% of A and 0% to 90% of B in 30 min. Fractions identified with the conjugate (absorbance at 260 nm) were lyophilized.

### 2.3. Chemical Characterization 

The UV-vis spectrum was determined in the wavelength range of 200 to 700 nanometers using a PerkinElmer Lambda Bio spectrophotometer (PerkinElmer, Waltham, MA, USA). The quartz cuvette was 1 cm in length. This spectrum was obtained using aqueous iFAP (0.2 mg/mL) and DOTA-iFAP (0.2 mg/mL) solutions. 

Infrared spectra of lyophilized powder samples of iFAP and DOTA-iFAP were obtained using the Fourier transform infrared (FT-IR) technique. The spectrum covered the range from 4000 to 400 cm². It was acquired by 40 scans with a resolution of 1 cm² using a PerkinElmer System 2000 spectrophotometer from Pike Technologies (PerkinElmer, Waltham, MA, USA). An attenuated total reflection (ATR) platform obtained the spectra. 

Mass spectra in 100 to 1500 mass units of charge (*m*/*z*) were generated using electrospray UPLC mass spectrometry (SQD-MS) for both iFAP and DOTA-iFAP lyophilized powders. These characterizations were achieved using an X bridge BEH C18 column (3.5 micron diameter, 4.6 mm width, and 150 mm length; Merck, Darmstadt, Hesse, Germany), ESI positive ion mode, 5V conical voltage, and Waters Mass Lynx 4.1 software (WatersCorp., Milford, MA, USA). Ten microliters of a 1 mg/mL concentrated solution were injected for analysis. An elution flow rate of 1 mL per minute was used with 0.1% TFA-acetonitrile (A) and 0.1% TFA-water (B) mobile phase. The gradients were linear at 100% A for 3 min, then switched to 50% A for 10 min, maintained at 50% A for another 10 min, switched to 30% A for 3 min, and finally switched back to 100% A for 4 min.

### 2.4. Preparation of ^68^Ga-iFAP

^68^Ga-iFAP was prepared using a synthesis module designed to label biomolecules with ^68^Ga (iQS^®^ Ga-68 Fluidic Labeling Module; ITM; Munich, Bavaria, Germany) for clinical use (GMP conditions). Briefly, 25 µg of DOTA-iFAP dissolved in 1 mL of 0.25 M sodium acetate, pH 4.5, was added to the system reactor and heated for 5 min (105–110 °C). Then, 5 mL of ^68^GaCl_3_ in 0.05 M HCl freshly eluted from the generator (^68^Ge/^68^Ga generator; ITM; Munich, Germany) was added and heated for 12 min (105–110 °C). Finally, it was purified using C18 Sep-Pak Light cartridges (solid phase extraction; Waters; Milford, MA, USA) and sterilized by a 0.22 µm membrane (Millipore; Burlington, MA, USA). The ^68^Ga-iFAP solution was assayed for radiochemical purity, sterility, and bacterial endotoxins following the general methods of the Mexican Pharmacopoeia [11].

### 2.5. Radiochemical Purity

Radiochemical purity was evaluated by reversed-phase radioactive HPLC (3.9 mm × 30 cm C18 column; Waters Corporation, Milford, MA, USA). An elution flow rate of 1 mL per minute was used with 0.1% TFA-acetonitrile (A) and 0.1% TFA-water (B) mobile phase. The gradients were linear at 100% A for 3 min, then switched to 50% A for 10 min, maintained at 50% A for another 10 min, switched to 30% A for 3 min, and finally switched back to 100% A for 4 min.

### 2.6. Stability

Samples were diluted (5×) in human serum (*n* = 3) and incubated at 37 °C to assess the stability of ^68^Ga-iFAP. Radiochemical purity was evaluated by size exclusion radioactive HPLC (column: ProteinPak 300SW; Waters Corporation, Milford, MA, USA) at 0.5 and 3 h.

### 2.7. Biological Characterization

#### 2.7.1. Cell Culture

In this study, the HCT116 cell line (ATCC^®^ CCL-247™; isolated from a human colorectal carcinoma; ATCC, Manassas, VA, USA) and AR42J pancreatic cancer cell line (ATCC^®^ CRL-CRL-1492™; isolated from a rat; ATCC, Manassas, VA, USA) were used for in vitro and in vivo ^68^Ga-iFAP evaluation.

RPMI-1640 medium (ATCC^®^ 30-2001™) provided by the ATCC (Manassas, VA, USA) was used to grow the cells in culture at 37 °C in an environment that contained 5% CO_2_. A 1% solution of penicillin and streptomycin (Thermo Fisher Scientific, Milford, MA, USA) and 10% fetal bovine serum (Merck, Darmstadt, Hesse, Germany) was added to the culture medium.

#### 2.7.2. Binding Assay

A competitive cell-binding test evaluated the binding affinity of ^68^Ga-iFAP. HCT116 cells (1 × 10^5^ cells/well) were placed in 96-well cell plates at 37 °C for 24 h. ^68^Ga-iFAP (70 µL; 7.4 kBq; 1 ng; 420 nmol/GBq) was added to each well, followed by the unlabeled DOTA-iFAP peptide (100 µL/well) at twelve concentrations from 10^−14^ to 10^−3^ M, and the wells were in the treatment for 1 h at 37 °C. Next, the liquid was decanted, and the cells were rinsed with PBS (*n* = 3). The amount of cell-bound radiotracer was calculated by measuring the radioactivity of a standard containing the initial ^68^Ga-iFAP activity (100%) and that of each well (% binding). The IC50 was determined using the Hill function to fit the competitive binding curve using Origin8 software (OriginLab, Northampton, MA, USA).

#### 2.7.3. Western Blot Assay

Cells were lysed by adding lysis buffer (CAT No. GB-181-100; Thermo Fisher Scientific, Hampton, NH, USA). After centrifugation at 20,000 × *g* for 15 min, SDS-PAGE was performed on 30 μg of protein per sample. Each gel was also run with a predetermined molecular weight standard (BLUEs-tainTM Protein Ladder 11-245 kDa Cat: P007-500). Proteins were then transferred to PVDF membranes (Merck Millipore, Burlington, MA, USA) and blocked with PBS containing 5% bovine serum albumin for 1 h at room temperature. Antibodies used for Western blotting were anti-β-actin peroxidase clone AC-15 (1:20,000 dilution; A3854, Merck Millipore, Burlington, MA, USA) and anti-FAP polyclonal Ab (rabbit 1:500 dilution; PA5-99458 Invitrogen: Waltham, MA, USA). After overnight incubation at 4 °C with primary antibodies, the membranes were washed. Membranes were incubated with species-specific HRP-conjugated secondary Ab for 1 h at room temperature, followed by extensive washing, incubation with Super Signal WestFem (Thermo Fisher Scientific, Milford, MA, USA), and detection using an Xtreme imaging system (Bruker, Billerica, MA, USA). In this system, the molecular weight marker image was obtained by reflectance, as the protein ladder used in this study is not luminescent. The detection bands of the proteins of interest (FAP and Actin) were obtained by luminescence imaging. Both image types were acquired simultaneously (Figure S1).

#### 2.7.4. Immunofluorescence

An immunofluorescence assay was performed to confirm the expression of FAP in BJ and AR42J cells. The cells were fixed with paraformaldehyde (4%) for 20 min, followed by Triton X-100 (0.5%) for permeabilization and 1% BSA for blocking. The cells were then incubated 12 h with a 1:50 polyclonal anti-FAP antibody (PA5-99458, Thermo Fisher Scientific, Milford, MA, USA), and then exposed to Alexa Fluor 488-conjugated goat anti-rabbit IgG (A32731, Thermo Fisher Scientific, Milford, MA, USA) for 1 h. DAPI (4′,6-diamidino-2-fenilindol; Merck, Darmstadt, Germany) was used to observe the intracellular fluorescence intensity of nuclear staining using a fluorescence microscope (MT6200, Meiji Techno.; Iruma, Saitama, Japan).

#### 2.7.5. Internalization and Uptake by Cancer Cells

^68^Ga-iFAP internalization and uptake by HCT116 cells (FAP-positive phenotype) and AR42J cells (FAP-negative phenotype) was determined using 1 × 10^6^ cells in PBS (500 µL) (*n* = 6). Both cell lines were placed with 10 µL of ^68^Ga-iFAP (18 kBq) for 1 h at 37 °C. After incubation, the cells were spun at 700 g for 5 min and washed three times with 500 µL of PBS in a 5415 R centrifuge (Eppendorf SE, Hamburg, Germany). A NaI (Tl) solid detector (MNL; Littleton, CO, USA) was used to assess the radioactivity of the cell pellet. A basal activity standard volume was used as a 100% reference, and cell activity was calculated relative to this. To assess ^68^Ga-iFAP internalization in the cells, pellets were incubated with 0.1 M glycine (500 µL) at pH 2.8 using 12 M HCl for 5 min at 37 °C and spun at 700 g for 5 min. The cellular sediment was treated with 1 M NaOH for 5 min and spun at 700 g for 5 min. Radioactivity in the liquid phase was quantified as the percent of internalization relative to intracellular activity. Uptake accounted for the remaining percentage.

#### 2.7.6. Biodistribution

All animal procedures were approved (protocol No. 10-2018-2022) under the Ethical Regulations for the Handling of Laboratory Animals (NOM-062-ZOO-1999) and the requirements of the Institutional Internal Committee for the Care and Use of Laboratory Animals (CICUAL-ININ). In the back, male Nu/Nu mice (6–8 weeks of age; CINVESTAV, I.PN., CDMX, Mexico City, Mexico) were subcutaneously injected with 1 × 10^6^ HCT116 cells/0.1 mL PBS. Tumor progression was monitored regularly at the injection sites. Animals were injected with 18.5 MBq (50 µL; 0.25 µg) of ^68^Ga-iFAP into the tail vein on day ten after tumor cell inoculation. They were sacrificed at 0.5, 1, and 3 h (*n* = 3). Blood samples were collected, and the kidney, lung, heart, liver, spleen, pancreas, small intestine, muscle, bone, and tumors were dissected. The radioactivity was measured by using a NaI(Tl) detector. Results were expressed as percent injected dose per gram tissue (%ID/g). To assess non-specific tumor uptake, a parallel biodistribution study at 1 h was performed in mice co-injected with 50 µL (50 µg) of iFAP (receptor blockade group of mice, I = 3). Tumors were analyzed by immunohistochemistry to evaluate FAP expression as detailed in Section 2.7.8.

#### 2.7.7. Infarcted Mouse Model Ex Vivo Imaging

Male Balb-c mice weighing between 35 and 45 g were used. The experimental procedure was developed according to protocol N° 10-2018-2022, adapted to the guidelines of the Ethical Regulations for the Handling of Laboratory Animals (NOM-062-ZOO-1999), and in accordance with the criteria of the Institutional Internal Committee for the Care and Use of Laboratory Animals (CICUAL-ININ). Myocardial infarction (acute FAP expression) was induced by preparing a solution of D, L-isoproterenol hydrochloride (Merck, Darmstadt, Hesse, Germany) dissolved in a 0.9% NaCl solution to a final concentration of 20 mg/mL. This solution was administered peritoneally at a dose of 100 mg/kg of body weight. Three hours after the injection of isoproterenol, 50 µL of a radiolabeled solution of ^68^Ga-iFAP (37 kBq) was administered through the tail vein. A group of three isoproterenol-treated mice and a control group (*n* = 3) that received 50 µL of 0.9% saline (isoproterenol-free) were sacrificed 30 min after radiopharmaceutical administration. A midline incision was made in the mice, and the heart was removed and conditioned in Petri dishes containing a PBS solution, followed by removal of the other organs (kidney, liver, lung, and spleen). Finally, an In-Vivo Xtreme preclinical imaging system (Bruker, Billerica, MA, USA) was used to obtain radioisotopic/x-ray/ex vivo images of mouse organs.

#### 2.7.8. Immunohistochemistry

HCCT tumors, healthy hearts (untreated mice), and isoproterenol-treated hearts (infarcted mice) were treated with paraformaldehyde (4%) at room temperature for 24 h, embedded in paraffin, and segmented (slices of 4–5 μm) using a Reichert-Jung microtome (Leica Biosystems, Lake Cook Road, IL, USA). Immunohistochemistry was conducted using deparaffinization, hydrating, and antigen retrieval. Slides were immersed in preheated citrate-EDTA solution ( pH 7.0, 2 mM EDTA, 10 mM citric acid, 0.05% Tween 2) at 100 °C for 30 min. This was followed by incubation with 2% hydrogen peroxide (H_2_O_2_) for 5 min and treated for 10 min with 5% BSA (endogenous peroxidase blocking). Samples were incubated overnight at 4 °C with FAP MoAb (PA5-99458, Thermo Fisher Scientific, Milford, MA, USA) diluted 1:100. Slides were washed and incubated with HRP-conjugated secondary donkey anti-rabbit IgG Ab (BioLegend: 406401; San Diego, CA, USA) at 1:100 dilution for 1 h and rinsed twice. Dako solution (Dako Liquid DAB+ Substrate Chromogen System: K3468; Agilent Technologies Inc., Santa Clara, CA, USA) was applied for 60 s to visualize immunoreactions after rinsing with Tris-buffered saline-Tween and distilled water for 5 min. Washing with PBS-Tween removed excess chromogen. Finally, they were counterstained with Harris hematoxylin, dehydrated, and coverslipped. Images were captured on an Axiostar microscope (Zeiss Group, Oberkochen, Stuttgart, Germany) using an INFINITY X-32 MP camera (Lumenera Corp., Ottawa, ON, Canada) in TIF format.

### 2.8. Clinical Imaging

This study (National Cancer Institute and Cardiology Ethics Committees: Protocol No. 2022-MN03) enrolled three healthy volunteers and three patients. Two patients were diagnosed with cancer (high-grade glioblastoma and breast cancer), and one had a recent myocardial infarction. The radiopharmaceutical micro-dose concept and proof-of-concept studies were the basis for the approval of the trial. Furthermore, the ethical standards of the Cancer and Cardiology Hospitals based on the Declaration of Helsinki regarding human experimentation and the GMP certificate granted to ININ by the Mexican Ministry of Health were considered. The volunteers and patients provided informed consent after receiving detailed information about the purpose of the study. The study could help evaluate a new radiopharmaceutical or decide on treatment and monitor the evolution of their disease. Patients underwent PET/CT imaging 30 min after the intravenous administration of ^68^Ga-iFAP (185 MBq) (Biograph 64 True Point PET/CT Scanner; Siemens, Charlottenburg, Berlin, Germany).

Three healthy volunteers were included (age range: 28–33 years; mean age ± SD, 30 ± 2.5 years; one male and two females). Subjects were excluded from the study if they had any clinical disease or surgery. A medical examination was performed and a medical history were taken.

Patients included: (1) A sixty-eight-year-old woman (weight 56 kg, height 156 cm) with a histopathology-confirmed diagnosis of infiltrating ductal carcinoma without a specific pattern, poorly differentiated with a Scarf Bloom Richardson (SBR) histologic scale value of 9 and grade 3 (SRB 9-G3) and with the presence of lymph vascular invasion. No perineural invasion was detected; triple-negative phenotype with Ki67 of 50%. (2) An eighty-year-old patient (53 kg, 180 cm tall) with a diagnosis of high-grade glioblastoma (glioblastoma WHO IV) and a previous cranial MRI (6 days prior) who underwent cranial and whole-body PET scanning. The whole-body scan also showed radiopharmaceutical uptake in a lesion associated with prostate cancer, subsequently confirmed by histopathology. (3) A sixty-year-old man with a medical history of smoking, alcoholism, type 2 diabetes mellitus, and systemic arterial hypertension of 20 years’ duration; no other cardiovascular history was reported. He presented with pressing chest pain on an 8/10 pain scale, radiating to the left arm and neck for 2 h, accompanied by headache, diaphoresis, and dyspnea. The next day, he presented with similar symptoms and was admitted to the emergency room. The basal electrocardiogram showed ST elevation in the anterior leads (V1–V4). Initial blood tests showed creatine kinase MB 255 UI/l, CK 2623, glucose 386 mg/dl, and NTPROBNP 2638. The diagnosis of anterior acute myocardial infarction with ST elevation and no reperfusion was included. A myocardial perfusion study was performed 3 days later with ^99m^Tc-sestamibi SPECT (^99m^Tc-MIBI; ININ, Ocoyoacac, Mexico) using a rest–stress protocol, as well as multiparametric cardiac magnetic resonance. ^68^Ga-iFAP/PET was acquired at 10, 30, and 60 min after radiotracer injection of the cardiac region with 10 min frames, each with low-dose CT attenuation correction.

## 3. Results and Discussion

### 3.1. Molecular Docking 

The affinity and inhibition constant (Ki) evaluation was performed by molecular docking of DOTA-D-Alanine-BoroPro compared with FAPI-04 (Table 1). It is known that ^68^Ga-labeled FAPI-04 has been evaluated in several cancers with FAP overexpression and has shown a higher detection sensitivity as well as a high tumor-to-noise ratio, even better than its ^18^F-labeled analogs [10,12,13,14]. For this reason, FAPI-04 was the control molecule in the docking process.

**Figure 1:**
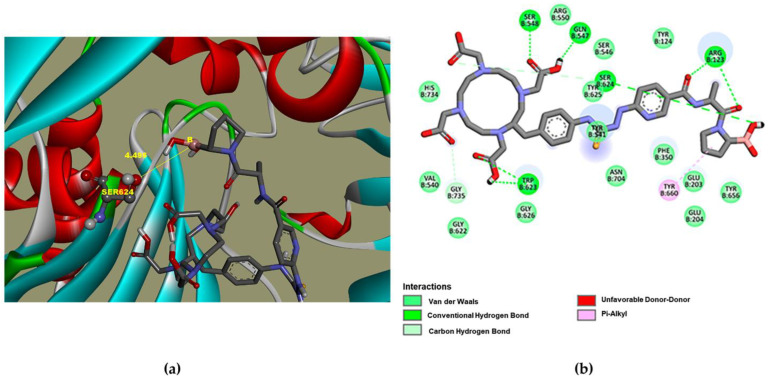
Molecular models: (**a**) FAP ligand-binding pocket for DOTA-iFAP, (**b**) DOTA-iFAP-FAP ligand binding pocket interaction map.

Figure 1 shows that DOTA-D-Alanine-BoroPro has a binding distance of 4.496 Å between the boron atom and Ser 624 and an affinity with an inhibition constant (Ki) of 18.02 nM. This affinity value can be attributed to the interaction of the hydrogen bridge of DOTA-D-Alanine-BoroPro with Arg 123. Since the amino acid Arg 123 is responsible for FAP having both endopeptidase and exopeptidase activities, an increase in selectivity towards DPPIV is generated. In addition, it should be noted that DOTA-D-Alanine-BoroPro has a more hydrophobic character in its structure, which favors the interaction with the hydrophobic S1 pocket entrance of the active site of FAP. It can be seen in the interactions with Tyr 625, Tyr 541, Trp 623, Gly 735, and Gly 626 [14]. These results support the possibility that the molecular structure of DOTA-iFAP has selective interactions and affinity for the FAP protein.

### 3.2. Chemical Synthesis

The chemical synthesis was carried out from HYNIC-iFAP using the methodology presented by Trujillo-Benítez et al. [7] Subsequently, Benzene-DOTA was added under the conditions shown in the methodology. The yield after HPLC purification was 80 ± 5%.

### 3.3. Chemical Evaluation

#### 3.3.1. DOTA-iFAP

**Figure 2:**
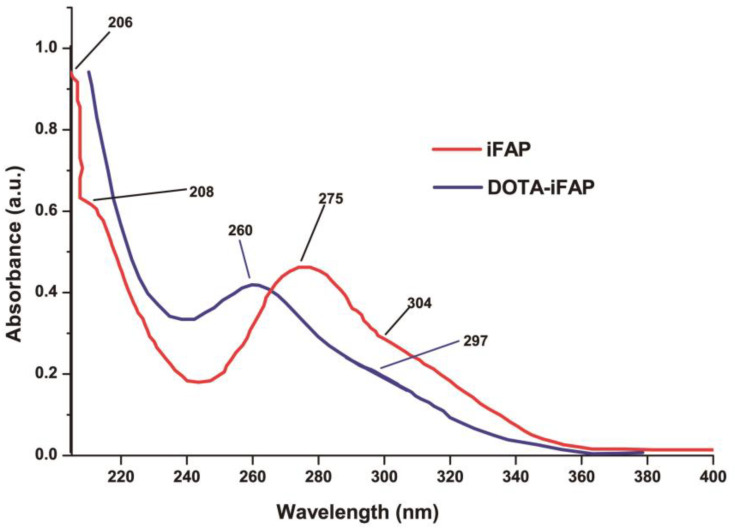
UV-vis spectra of iFAP and benzene-DOTA-iFAP (DOTA-iFAP).

The iFAP UV-vis spectrum (Figure 2) shows bands at 206 nm and 208 nm, related to hydrazine (C-NH-NH_2_) n → σ* transitions. These bands disappear in DOTA-iFAP (Figure 2) due to the interaction between the N of hydrazine and the carbonyl of benzene-DOTA. Also, the band at 260 nm associated with the n → π* transitions (-C=S-N- groups) appears. The shoulder at 297 nm is associated with the pyridine ring’s n→π* transitions (C=N- group).

The spectrum from the reversed-phase HPLC analysis performed at the end of the reaction shows the presence of iFAP (placed in excess) with a retention time (t_R_) of 10.2 min and DOTA-iFAP (t_R_ = 11.5 min), which verified that the conjugation reaction was carried out. The difference between the retention times allowed for the adequate HPLC purification of the DOTA-iFAP conjugate using a C-18 preparative column.

**Figure 3:**
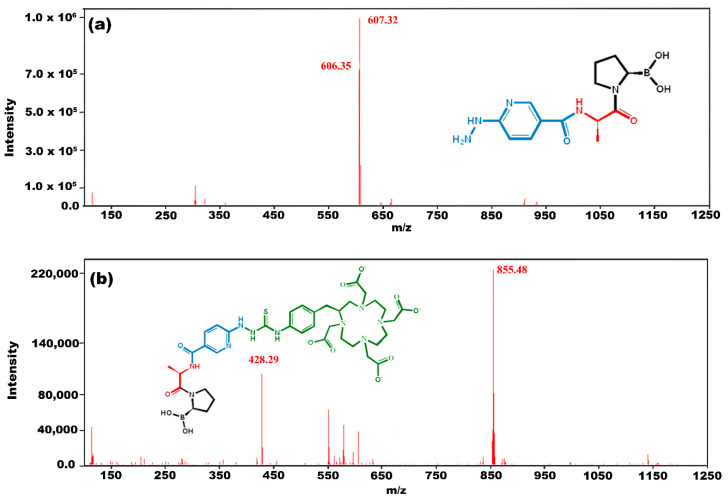
Mass spectra of (**a**) iFAP and (**b**) benzene-DOTA-iFAP (DOTA-iFAP).

The mass spectrum from HPLC for the iFAP peak (t_R_ = 10.2 min) showed corresponding signal with *m*/*z* 322 (cal. 321) [M+H]+ and *m*/*z* 606 2×[M+H-H_2_O]^+^ (Figure 3a), while the mass spectrum from HPLC for the DOTA-iFAP peak (t_R_ = 11.5 min) showed corresponding signals with *m*/*z* 855.48 (cal 872) [M+H-H_2_O] and *m*/*z* 428.29 [M+H-H_2_O]/2. The same spectrum was observed for the purified lyophilized DOTA-iFAP product (Figure 3b).

**Figure 4:**
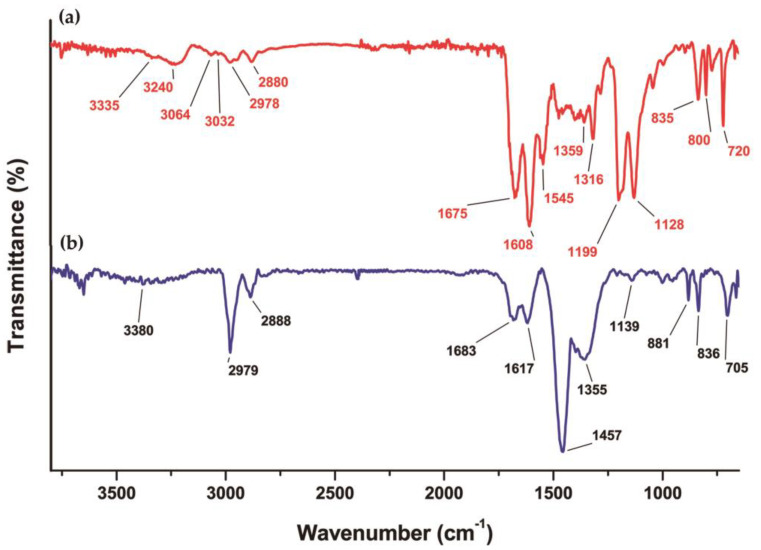
Infrared spectra of (**a**) iFAP and (**b**) benzene-DOTA-iFAP (DOTA-iFAP).

In the FT-IR spectrum of iFAP, bands at 3335 cm^−1^, 1675 cm^−1^, 1128 cm^−1^, and 1199 cm^−1^ corresponding to the N-H vibrations (from hydrazine) were observed (Figure 4a). These bands disappeared in the DOTA-iFAP spectrum; the bands corresponding to N-H (1683 cm^−1^ and 1617 cm^−1^) appeared in their place. Furthermore, a new band was observed at the 1457 cm^−1^ signal, assigned to the -C=O groups of the DOTA ring (Figure 4b). The bands corresponding to the B-O vibration (1359–1355 cm^−1^) and those corresponding to the υs-CH_2_ and υas-CH_2_ (2989 cm^−1^–2880 cm^−1^) were observed in both spectra.

#### 3.3.2. ^68^Ga-iFAP Radiochemical Purity

**Figure 5:**
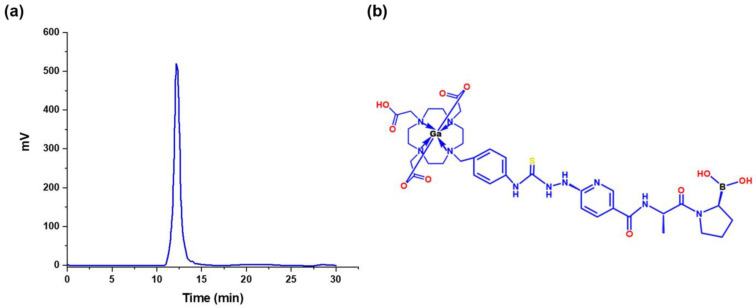
(**a**) Radiochromatogram of ^68^Ga-iFAP (C18 column, 3.9 × 300 mm, 10 µm particle size). (**b**) Schematic structure of the ^68^Ga-iFAP radiocomplex.

Radio-HPLC analysis of the DOTA-iFAP radiolabeled with ^68^Ga showed a radiochemical yield of 76.2 ± 5.2% and a radiochemical purity greater than 95% (Figure 5) with a retention time of 12.17 min. The difference between the retention times of DOTA-iFAP (11.5 min) and ^68^Ga-DOTA-iFAP (^68^Ga-iFAP) (12.17 min) is because the tubing volume between the radio detector and the UV-vis detector is 0.7 mL, producing a difference between the chromatograms of 0.7 min at the flow rate of 1 mL/min.

#### 3.3.3. ^68^Ga-iFAP Stability

^68^Ga-iFAP was placed in human serum at 37 °C for 30 min and the radiocomplex remained stable (radiochemical purity > 97% at 3 h). The chromatographic profiles (radiochromatogram and UV-vis chromatogram) indicated the presence of proteins between 4 and 7 min and a retention time for ^68^Ga-iFAP and ^68^GaCl_3_ of 8.3 min and 12 min, respectively. The radioactivity associated with the protein retention time (4–7 min) was the percent of radiopharmaceutical binding to proteins when analyzing the samples at different time points. Therefore, in vitro ^68^Ga-iFAP stability in serum showed a binding to proteins of 0.5 ± 0.2% at 30 min and 0.8 ± 0.2% at 3 h, as well as a high radiochemical stability (>97% at 3 h).

### 3.4. Biological Characterization 

#### 3.4.1. Binding Assay

**Figure 6:**
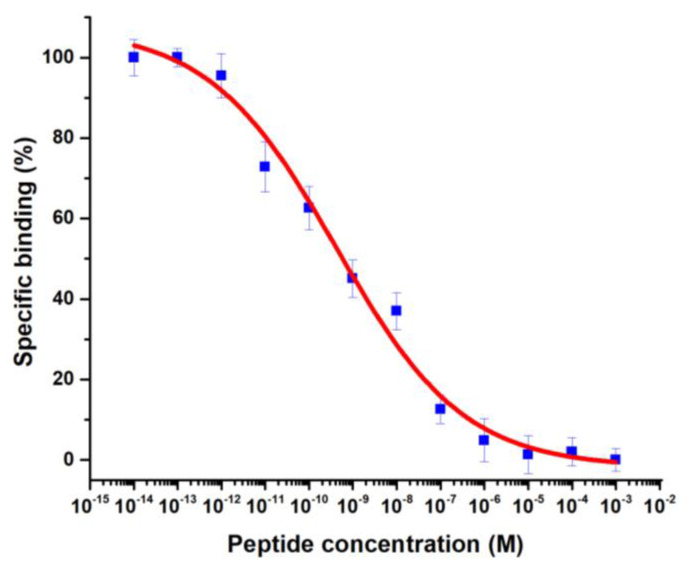
Competition binding assay of ^68^Ga-iFAP (420 nmol/GBq) with the unlabeled peptide (DOTA-iFAP) at 12 concentrations in HCT116 cells (1 × 10^5^ cells/well). ^68^Ga-iFAP IC_50_ = 5.7 × 10^−10^ M (0.57 nM).

The competition binding assay of ^68^Ga-iFAP with the unlabeled peptide (DOTA-iFAP) in HCT116 cells showed a ^68^Ga-iFAP IC_50_ of 5.7 × 10^−10^ M (0.57 nM) (Figure 6). The IC50 value indicated good affinity for the FAP protein compared with that of ^68^Ga-FAPI-04 (IC_50_ = 6.5 nM) or ^68^Ga-FAPI-13 (IC_50_ = 4.5 nM) [15]. Nevertheless, for a real comparison, binding assays should be performed under the same experimental conditions. 

#### 3.4.2. Immunofluorescence, Western Blot, Cellular Uptake, and Internalization

**Figure 7:**
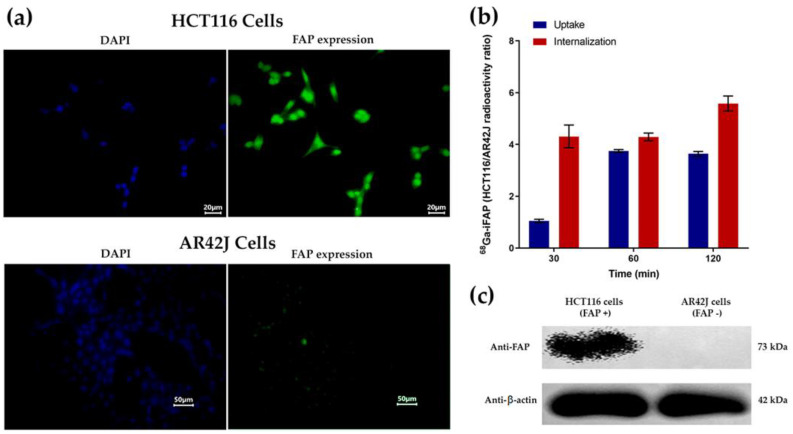
(**a**) Micrographs of immunofluorescence staining for FAP in HCT116 cells, demonstrating positive FAP expression, and in AR42J cells with low FAP expression (FAP stained in green with anti-FAP and cell nuclei stained in blue with DAPI). (**b**) Kinetics of cellular uptake and internalization of ^68^Ga-iFAP in HCT116 cells expressed as an HCT116/AR42J radioactivity ratio. (**c**) Western blot assay demonstrating FAP expression in HCT116 cells and low expression in AR42J cells (luminescence images). FAP and actin molecular weight proteins correlated with the protein ladder used as molecular weight marker reference (Figure S1).

The expression of FAP in HCT116 cells (FAP stained in green with anti-FAP and nuclei stained in blue with DAPI) and the low FAP expression in AR42J cells (Figure 7a) were confirmed by immunofluorescence microscopy and Western blot (Figure 7c), (Figure S1) ensuring that ^68^Ga-iFAP uptake is specific and related with the expression of FAP in HCT116 cells. In the uptake and internalization kinetics (Figure 7b), it was observed that HCT116 cells (FAP-positive control) took up the ^68^Ga-iFAP radiopharmaceutical with rapid internalization. That is, after 30 min of incubation, the ratio of ^68^Ga-iFAP uptake to AR42J cells was close to 1 because all of the ^68^Ga-iFAP taken up by HCT116 cells had already been internalized (HCT116/AR42J internalization ratio greater than 4) (Figure 7b). The uptake and internalization of ^68^Ga-iFAP by AR42J cells was not significantly different from the background signal. After 60 min, the amount of ^68^Ga-iFAP in the HCT116 membrane (cell uptake) was significantly increased (almost four times more than in AR42J cells), associated with the time of maximum cell uptake, of which a significant amount was internalized after 120 min (Figure 7b). Lindner et al. also demonstrated a fast and significant internalization of ^68^Ga-FAPI-01 and ^68^Ga-FAPI-02 from 30 to 60 min, which was attributed to a dynamin-dependent endocytosis process [16]. Therefore, ^68^Ga-iFAP specifically recognizes the FAP protein, as demonstrated in this assay, which correlated with the iFAP-specific recognition [7].

#### 3.4.3. Biodistribution

**Figure 8:**
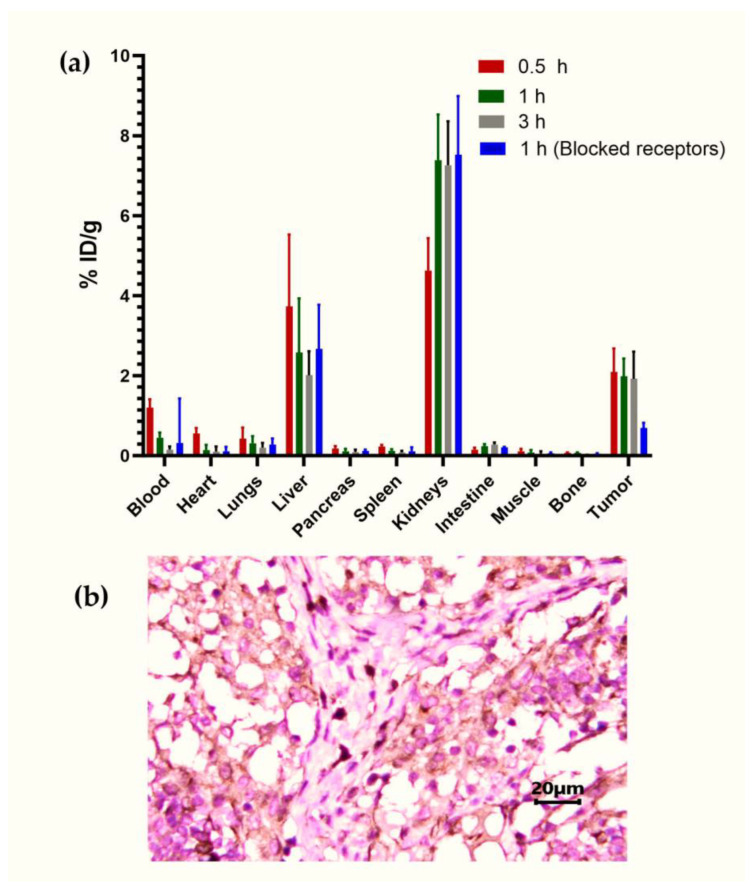
(**a**) Biodistribution of ^68^Ga-iFAP in mice bearing HCT116 colon cancer tumors at 0.5, 1 (with blocked and unblocked receptors), and 3 h after administration. (**b**) Immunohistochemistry micrography of the ex vivo HCT116 tumor demonstrating FAP expression (brown color).

The evaluation of the ^68^Ga-iFAP biodistribution in nude mice with induced lung cancer tumors (HCT116 cells) showed a tumor uptake of 2.09 ± 0.59% of the administered activity per gram of tissue (% ID/g) at 30 min after injection with a slight decrease at 1 and 2 h (1.98 ± 0.45% and 1.92 ± 0.68%, respectively). The extremely rapid clearance was mainly via the renal route (Figure 8a). The biokinetics in the primary target organs (liver and kidney) or tissues (blood and tumor) of the radiopharmaceutical are also shown in Figure 8a. The results sowed a rapid elimination in the blood, which correlated with the results found for the distribution of other FAPIs [7,17]. In the receptor blockade group of mice, tumor uptake was 0.69 ± 0.13% ID/g, which was significantly lower (*p* < 0.05) compared to that in the unblocked group (Figure 8a). Immunohistochemistry analysis of the ex vivo tumor showed FAP expression, which can be associated with the specific ^68^Ga-iFAP tumor uptake (Figure 8b). In addition, the radioligand administration showed no adverse effects when administered to laboratory mice.

#### 3.4.4. Infarcted Mouse Model Ex Vivo Imaging

**Figure 9:**
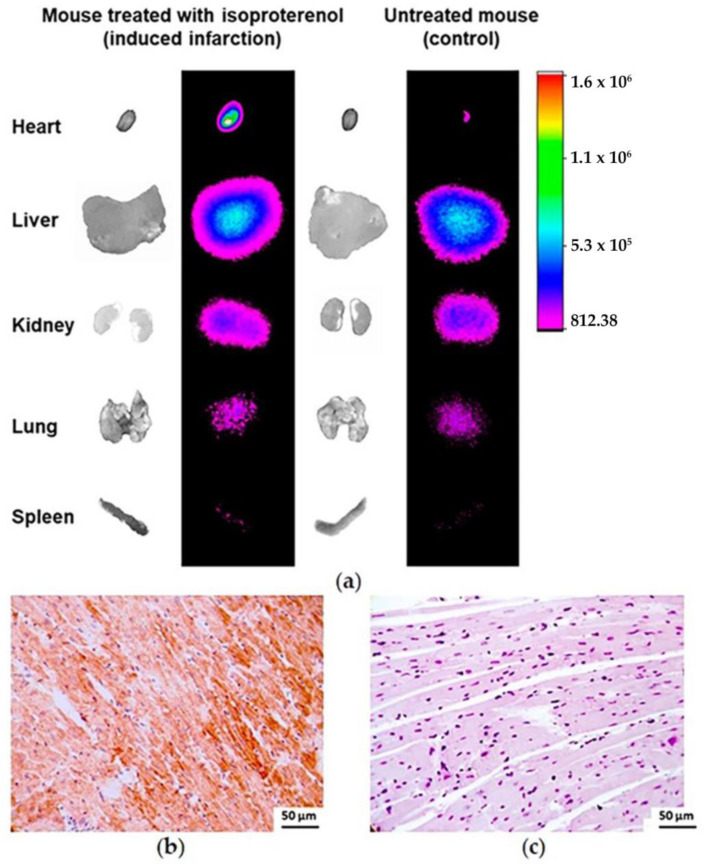
(**a**) X-ray/radioisotopic/ex vivo images of ^68^Ga-iFAP uptake in an infarcted (isoproterenol-treated) mouse model (left) and in an untreated mouse (right) 0.5 h after radiotracer administration. Immunohistochemistry photomicrography (400×) of a heart from (**b**) an isoproterenol-treated mouse with a high level of FAP expression (brown color) and (**c**) an untreated mouse (with no overexpression of FAP).

Isoproterenol-treated mice exhibited high cardiac uptake of ^68^Ga-iFAP compared to control hearts, as demonstrated by radioisotopic/x-ray/ex vivo images (Figure 9a). The high radiotracer uptake was associated with the induction of FAP expression in response to isoproterenol-induced myocardial tissue damage, as confirmed by immunohistochemistry (Figure 9b). As can be seen, FAP expression (brown color) in the mouse heart is observed only in the treated group (induced infarction), since, under normal conditions, the heart does not overexpress FAP, as found in the control group (Figure 9b). This finding confirms the potential of ^68^Ga-iFAP to determine the risk of excessive fibrosis after myocardial infarction because reactive fibroblasts modulate cardiac fibrosis and, consequently, post-infarction tissue remodeling [18,19,20]. Therefore, ^68^Ga-iFAP may provide information on the progression of fibrosis and allow for an estimation of patient survival after myocardial infarction, as has been proposed for other FAPI derivatives [10,21,22].

### 3.5. Clinical Imaging

**Figure 10:**
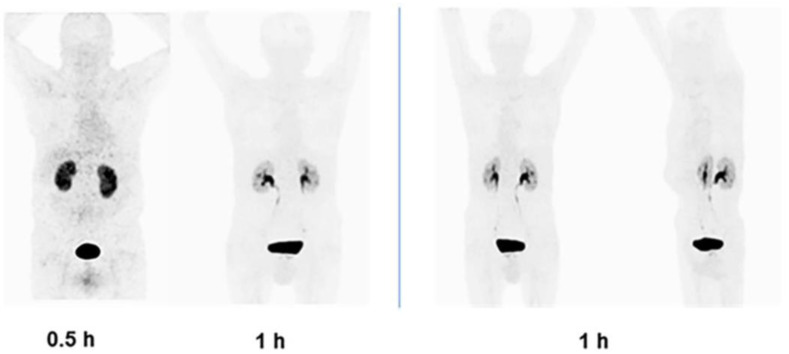
^68^Ga-iFAP/PET images (anterior and lateral) of healthy subjects at 0.5 h, and 1 h after radiotracer intravenous administration (185 MBq).

No adverse events were observed in patients or volunteers related to the diagnostic use of ^68^Ga-iFAP. Physiological radioligand uptake (blood pool, kidneys, and, to a lesser degree, the liver) was visualized in the body scan of healthy volunteers 30 min after radiotracer administration. Biliary and renal elimination was observed (Figure 10). After 1 h, the ^68^Ga-iFAP was cleared from the whole body and remained only in the metabolic organs, mainly in the kidney (Figure 10). The rapid elimination of ^68^Ga-iFAP was correlated with that reported for other FAPI/PET radiotracers [23,24,25].

**Figure 11:**
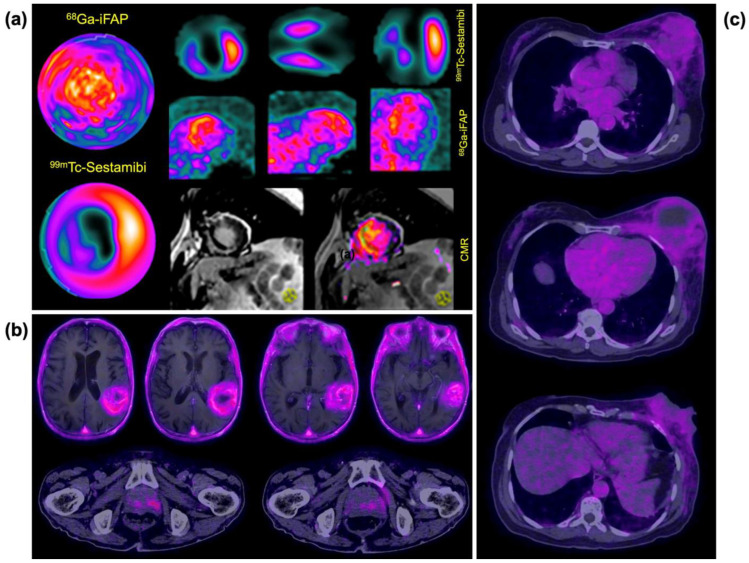
(**a**) ^68^Ga-iFAP/PET, ^99m^Tc-Sestamibi/SPECT, and cardiac magnetic resonance (CMR) imaging in a patient with acute myocardial infarction. Note the high ^68^Ga-iFAP uptake in the anteroseptal region corresponding to the transmural infarct area of the culprit lesion. ^68^Ga-iFAP uptake in histologically confirmed primary tumors of (**b**) high-grade glioblastoma in the left temporoparietal region (top) coexisting with prostate cancer (bottom) and (**c**) triple-negative left breast cancer with a large central necrotic component.

The images of patients diagnosed with acute myocardial infarction showed high ^68^Ga-iFAP uptake in the anteroseptal region corresponding to the transmural infarct area of the culprit lesion (Figure 11a). ^99m^Tc-sestamibi SPECT images acquired using a rest–stress protocol showed a transmural infarct in the apex and anteroseptal region. It is important to note that ^99m^Tc-MIBI only assesses myocardial perfusion, limiting its visualization to the periphery of the infarct area. In contrast, ^68^Ga-iFAP accumulates specifically within the infarct zone due to FAP expression. Therefore, both systems are expected to have synergistic utility in cardiology. Multiparametric cardiac magnetic resonance (CMR) visualized late gadolinium enhancement (LGE) in the anteroseptal region, also demonstrating a transmural infarct (Figure 11a). Microvascular obstruction was also present at these sites. Recently, Diekmann et al. presented 35 patients who underwent multi-modality cardiac imaging, including ^68^Ga-FAPI-46 PET, within 11 days of reperfusion therapy for acute myocardial infarction. They confirmed that regional upregulation of FAP by activated fibroblasts showed a very high contrast between the injured infarct and peri-infarct regions. Also, FAP upregulation exceeded the infarct region, which could be explained by reactive fibrosis that may compromise non-infarcted myocardium [26].

Significant uptake of ^68^Ga-iFAP was observed in histologically confirmed primary tumors of high-grade glioblastoma (Figure 11b, top), prostate cancer (Figure 11b, bottom), and triple-negative breast cancer (Figure 11c). 

FAP is heavily involved in tissue remodeling and promotes tumorigenesis through multiple mechanisms, including angiogenesis, with the promotion of surrounding tissue invasion, immune suppression, epithelial–mesenchymal transition, drug resistance, and stem cell promotion [27]. It has also been shown that FAP overexpression is associated with lymphocyte-dependent immune responses [28]. Therefore, the evaluation of FAP overexpression by ^68^Ga-iFAP/PET or ^99m^Tc-iFAP/SPECT in primary or metastatic tumors will allow clinicians to determine whether the patient will benefit from immune checkpoint inhibitor (ICI) therapies, treatment with FAP-based targeted radiotherapy such as ^177^Lu-FAPI [29] or ^177^Lu-iFAP/^177^Lu-iPSMA-based dual-targeted colloidal systems [30], or, as an optimal option, the combination of immunotherapy and targeted radiotherapy, as well as future therapies based on FAP inhibitors for the treatment of aggressive cancers.

## 4. Conclusions

The ^68^Ga-DOTA-D-Alanine-BoroPro (^68^Ga-iFAP) radiotracer shows suitable properties as a novel radiotracer for PET/FAP imaging in both malignant and non-malignant tissues. Analysis results indicate high radiotracer stability in human serum, in vitro and in vivo specificity for FAP, and rapid renal clearance in humans. The findings of this research justify further dosimetry and clinical trials to establish the specificity and sensitivity of 68Ga-iFAP PET for FAP expression imaging.

## Figures and Tables

**Table 1:** Fibroblast Activation Protein (FAP) affinities, Ki’s, and boron/serine-624 interaction distances evaluated by molecular docking.

Ligand	Affinity (kcal/mol)	*Ki* (nM)	Distance Ser624–Boro (Å)
**FAPI-O4** 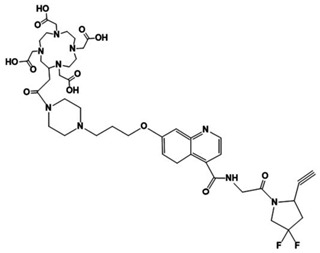	−10.56	18.12	4.29
**DOTA-D-Alanine-BoroPro (DOTA-iFAP)** 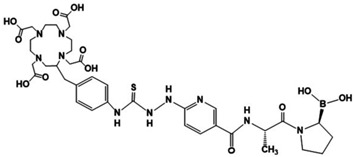	−10.89	18.02	4.49

## Data Availability

Data are contained within the article.

## Supplementary Materials

The following supporting information can be downloaded at: https://www.mdpi.com/article/10.3390/pharmaceutics16040532/s1, Figure S1: Full Western Blot membranes for (a) Actin and (b) FAP protein of HCT116 and AR42J cells. In this system, the molecular weight marker image was obtained by reflectance, as the protein ladder used in this study is not luminescent (BLUEstainTM Protein Ladder 11–245 kDa Cat: P007-500). The detection bands of the proteins of interest (FAP and Actin) were obtained by luminescence imaging (HRP chemiluminescent reaction). Both image types were acquired simultaneously in a Xtreme preclinical imaging system (Bruker, Billerica, MA, USA).

## References

[1] Altmann A., Haberkorn U., Siveke J. (2021). The latest developments in imaging of fibroblast activation protein. J. Nucl. Med..

[2] Fitzgerald A.A., Weiner L.M. (2020). The role of fibroblast activation protein in health and malignancy. Cancer Metastasis Rev..

[3] Li M., Younis M.H., Zhang Y., Cai W., Lan X. (2022). Clinical summary of fibroblast activation protein inhibitor-based radiopharmaceuticals: Cancer and beyond. Eur. J. Nucl. Med. Mol. Imaging.

[4] Xin L., Gao J., Zheng Z., Chen Y., Lv S., Zhao Z., Yu C., Yang X., Zhang R. (2021). Fibroblast activation protein-α as a target in the bench-to-bedside diagnosis and treatment of tumors: A narrative review. Front. Oncol..

[5] Liu Y., Watabe T., Kaneda-Nakashima K., Shirakami Y., Naka S., Ooe K., Toyoshima A., Nagata K., Haberkorn U., Kratochwil C. (2022). Fibroblast activation protein targeted therapy using [177 Lu] FAPI-46 compared with [225 Ac] FAPI-46 in a pancreatic cancer model. Eur. J. Nucl. Med. Mol. Imaging.

[6] Luo X., Zhang Z., Cheng C., Wang T., Fang D., Zuo C., Yuan G., Li R., Li X. (2023). SPECT Imaging with Tc-99m-Labeled HYNIC-FAPI-04 to Extend the Differential Time Window in Evaluating Tumor Fibrosis. Pharmaceuticals.

[7] Trujillo-Benítez D., Luna-Gutiérrez M., Ferro-Flores G., Ocampo-García B., Santos-Cuevas C., Bravo-Villegas G., Morales-Ávila E., Cruz-Nova P., Díaz-Nieto L., García-Quiroz J. (2022). Design, Synthesis and Preclinical Assessment of 99mTc-iFAP for In Vivo Fibroblast Activation Protein (FAP) Imaging. Molecules.

[8] Alqahtani F.F. (2022). SPECT/CT and PET/CT, related radiopharmaceuticals, and areas of application and comparison. Saudi Pharm. J..

[9] Mori Y., Dendl K., Cardinale J., Kratochwil C., Giesel F.L., Haberkorn U. (2023). FAPI PET: Fibroblast activation protein inhibitor use in oncologic and nononcologic disease. Radiology.

[10] Varasteh Z., Mohanta S., Robu S., Braeuer M., Li Y., Omidvari N., Topping G., Sun T., Nekolla S.G., Richter A. (2019). Molecular imaging of fibroblast activity after myocardial infarction using a 68Ga-labeled fibroblast activation protein inhibitor, FAPI-04. J. Nucl. Med..

[11] (2021). Pharmacopoeia of the United Mexican States (Thirteenth Edition), Mexico. https://www.farmacopea.org.mx/publicaciones-detalle.php?m=3&pid=12..

[12] Hu K., Li J., Wang L., Huang Y., Li L., Ye S., Han Y., Huang S., Wu H., Su J. (2022). Preclinical evaluation and pilot clinical study of [18F] AlF-labeled FAPI-tracer for PET imaging of cancer associated fibroblasts. Acta Pharm. Sin. B.

[13] Wang H., Zhu W., Ren S., Kong Y., Huang Q., Zhao J., Guan Y., Jia H., Chen J., Lu L. (2021). 68Ga-FAPI-04 versus 18F-FDG PET/CT in the detection of hepatocellular carcinoma. Front. Oncol..

[14] Poplawski S.E., Lai J.H., Li Y., Jin Z., Liu Y., Wu W., Wu Y., Zhou Y., Sudmeier J.L., Sanford D.G. (2013). Identification of selective and potent inhibitors of fibroblast activation protein and prolyl oligopeptidase. J. Med. Chem..

[15] Lindner T., Loktev A., Altmann A., Giesel F., Kratochwil C., Debus J., Jäger D., Mier W., Haberkorn U. (2018). Development of quinoline-based theranostic ligands for the targeting of fibroblast activation protein. J. Nucl. Med..

[16] Lindner T., Loktev A., Giesel F., Kratochwil C., Altmann A., Haberkorn U. (2019). Targeting of activated fibroblasts for imaging and therapy. EJNMMI Radiopharm. Chem..

[17] Chen H., Pang Y., Wu J., Zhao L., Hao B., Wu J., Wei J., Wu S., Zhao L., Luo Z. (2020). Comparison of [68 Ga] Ga-DOTA-FAPI-04 and [18 F] FDG PET/CT for the diagnosis of primary and metastatic lesions in patients with various types of cancer. Eur. J. Nucl. Med. Mol. Imaging.

[18] Xie B., Wang J., Xi X.-Y., Guo X., Chen B.-X., Li L., Hua C., Zhao S., Su P., Chen M. (2022). Fibroblast activation protein imaging in reperfused ST-elevation myocardial infarction: Comparison with cardiac magnetic resonance imaging. Eur. J. Nucl. Med. Mol. Imaging.

[19] Tillmanns J., Hoffmann D., Habbaba Y., Schmitto J.D., Sedding D., Fraccarollo D., Galuppo P., Bauersachs J. (2015). Fibroblast activation protein alpha expression identifies activated fibroblasts after myocardial infarction. J. Mol. Cell. Cardiol..

[20] Ma Y., Iyer R.P., Jung M., Czubryt M.P., Lindsey M.L. (2017). Cardiac fibroblast activation post-myocardial infarction: Current knowledge gaps. Trends Pharmacol. Sci..

[21] Notohamiprodjo S., Nekolla S.G., Robu S., Villagran Asiares A., Kupatt C., Ibrahim T., Laugwitz K.-L., Makowski M.R., Schwaiger M., Weber W.A. (2021). Imaging of cardiac fibroblast activation in a patient after acute myocardial infarction using 68 Ga-FAPI-04. J. Nucl. Cardiol..

[22] Zhang M., Quan W., Zhu T., Feng S., Huang X., Meng H., Du R., Zhu Z., Qu X., Li P. (2023). [68Ga] Ga-DOTA-FAPI-04 PET/MR in patients with acute myocardial infarction: Potential role of predicting left ventricular remodeling. Eur. J. Nucl. Med. Mol. Imaging.

[23] Loktev A., Lindner T., Burger E.-M., Altmann A., Giesel F., Kratochwil C., Debus J., Marmé F., Jäger D., Mier W. (2019). Development of fibroblast activation protein–targeted radiotracers with improved tumor retention. J. Nucl. Med..

[24] Dong Y., Zhou H., Alhaskawi A., Wang Z., Lai J., Yao C., Liu Z., Hasan Abdullah Ezzi S., Goutham Kota V., Hasan Abdulla Hasan Abdulla M. (2023). The Superiority of Fibroblast Activation Protein Inhibitor (FAPI) PET/CT Versus FDG PET/CT in the Diagnosis of Various Malignancies. Cancers.

[25] Nader M., Valla D., Vriamont C., Masset J., Pacelli A., Herrmann K., Zarrad F. (2022). [68Ga]/[90Y] FAPI-46: Automated production and analytical validation of a theranostic pair. Nucl. Med. Biol..

[26] Diekmann J., Koenig T., Thackeray J.T., Derlin T., Czerner C., Neuser J., Ross T.L., Schäfer A., Tillmanns J., Bauersachs J. (2022). Cardiac fibroblast activation in patients early after acute myocardial infarction: Integration with MR tissue characterization and subsequent functional outcome. J. Nucl. Med..

[27] Kalaei Z., Manafi-Farid R., Rashidi B., Kiani F.K., Zarei A., Fathi M., Jadidi-Niaragh F. (2023). The Prognostic and therapeutic value and clinical implications of fibroblast activation protein-α as a novel biomarker in colorectal cancer. Cell Commun. Signal..

[28] Liao Y., Ni Y., He R., Liu W., Du J. (2013). Clinical implications of fibroblast activation protein-α in non-small cell lung cancer after curative resection: A new predictor for prognosis. J. Cancer Res. Clin. Oncol..

[29] Baum R.P., Schuchardt C., Singh A., Chantadisai M., Robiller F.C., Zhang J., Mueller D., Eismant A., Almaguel F., Zboralski D. (2022). Feasibility, biodistribution, and preliminary dosimetry in peptide-targeted radionuclide therapy of diverse adenocarcinomas using 177Lu-FAP-2286: First-in-humans results. J. Nucl. Med..

[30] Luna-Gutiérrez M., Ocampo-García B., Jiménez-Mancilla N., Ancira-Cortez A., Trujillo-Benítez D., Hernández-Jiménez T., Ramírez-Nava G., Hernández-Ramírez R., Santos-Cuevas C., Ferro-Flores G. (2022). Targeted Endoradiotherapy with Lu_2_O_3_-iPSMA/-iFAP Nanoparticles Activated by Neutron Irradiation: Preclinical Evaluation and First Patient Image. Pharmaceutics.

