# Supplementary material for: 68Ga-DOTA-D-Alanine-BoroPro Radiotracer for Imaging of the Fibroblast Activation Protein in Malignant and Non-Malignant Diseases
Source: Pharmaceutics. 2024 Apr 12;16(4):532. doi: 10.3390/pharmaceutics16040532 (PMC11054143; doi:10.3390/pharmaceutics16040532)
Supplement: Supplementary file 1 [file pharmaceutics-16-00532-s001.zip › pharmaceutics-2841493-supplementary.pdf]

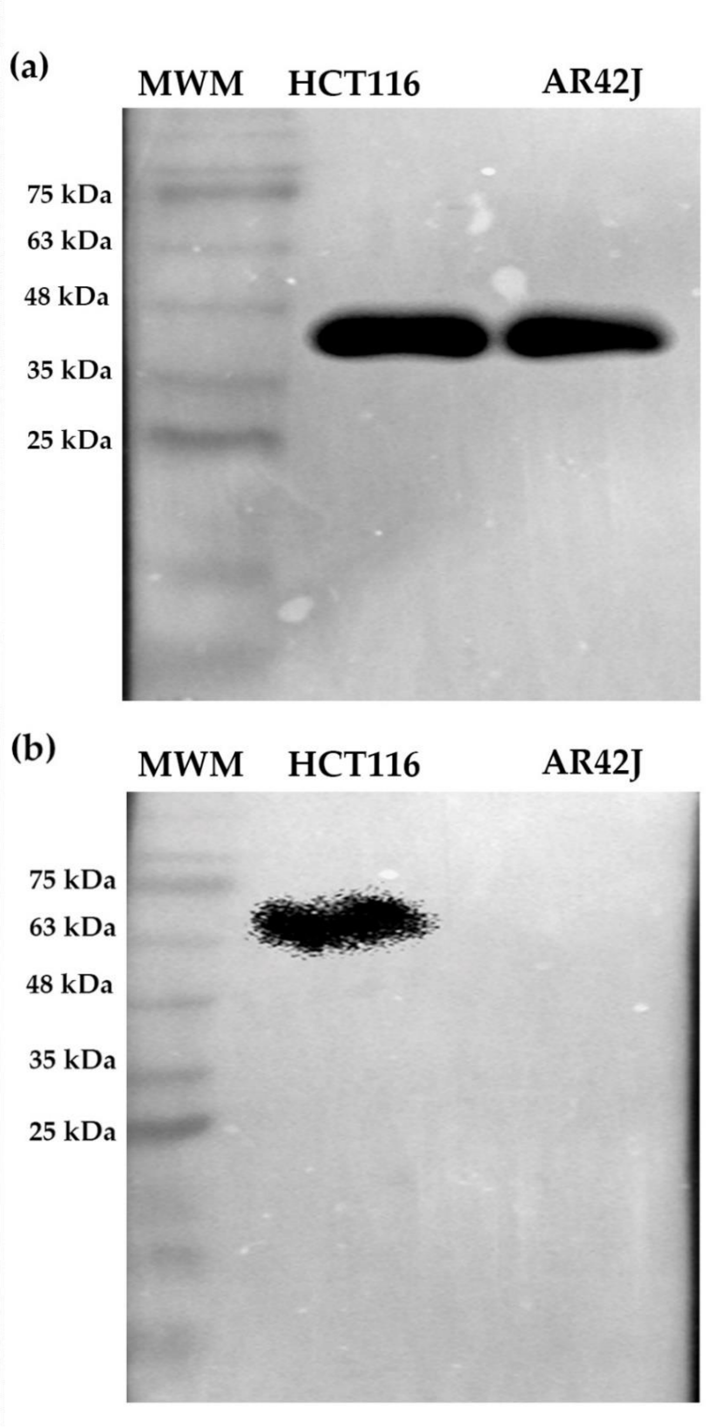

Figure S1. Full Western Blot membranes for (a) Actin and (b) FAP protein of HCT116 and AR42J cells. In this system, the molecular weight marker image was obtained by reflectance, as the protein ladder used in this study is not luminescent (BLUEstainTM Protein Ladder 11–245 kDa Cat: P007-500). The detection bands of the proteins of interest (FAP and Actin) were obtained by luminescence imaging (HRP chemiluminescent reaction). Both image types were acquired simultaneously in a Xtreme preclinical imaging system (Bruker, Billerica, MA, USA).
